# Supplementary material for: Inference of glioblastoma migration and proliferation rates using single time-point images
Source: Commun Biol. 2023 Apr 13;6:402. doi: 10.1038/s42003-023-04750-0 (PMC10102065; doi:10.1038/s42003-023-04750-0)
Supplement: Supplementary file 2 — Supplementary Figures [file 42003_2023_4750_MOESM2_ESM.pdf]

Supplemental Figures

Inference of glioblastoma migration and proliferation rates using single time-point images,

Rosén et al.

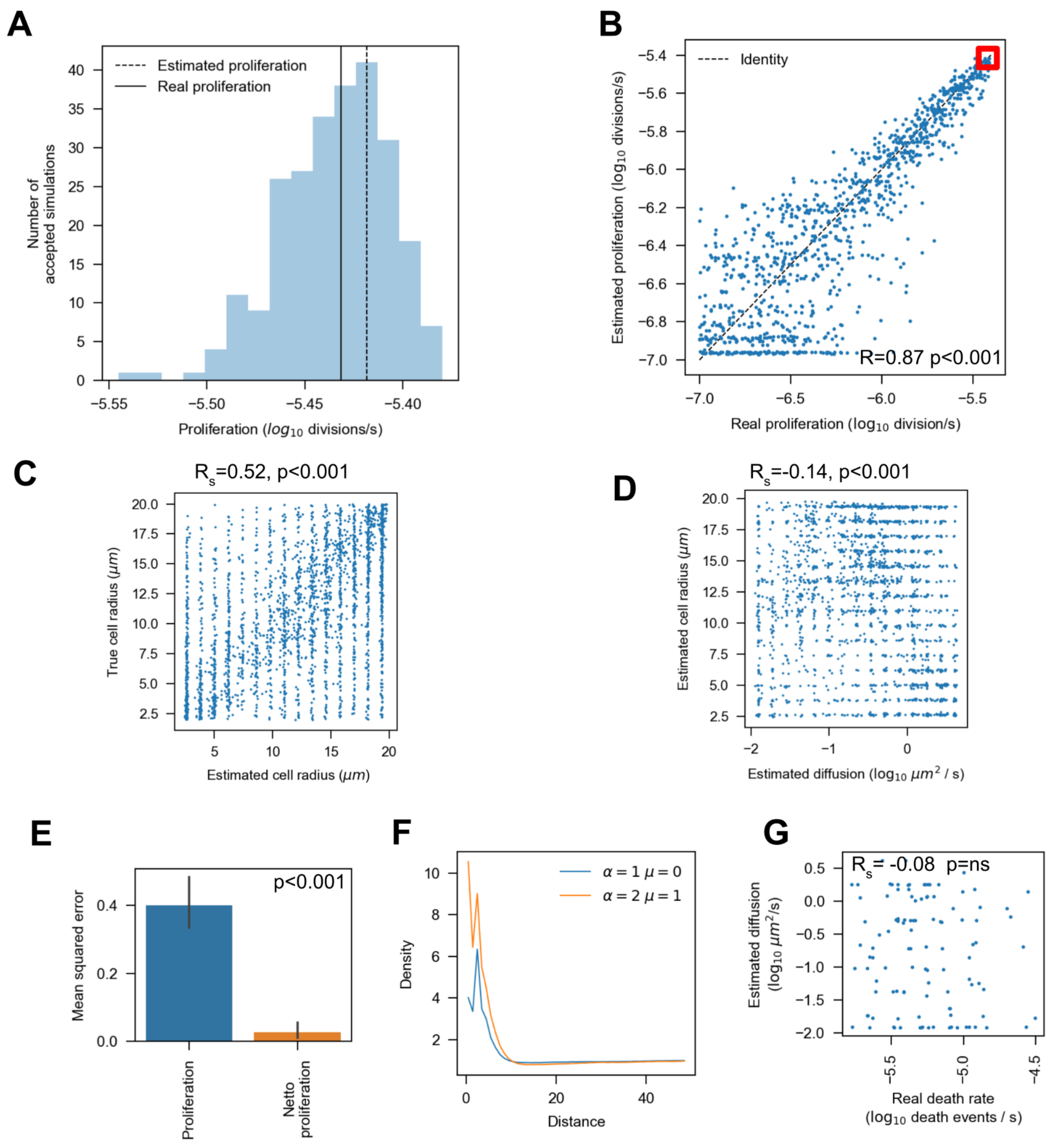

Supplemental Figure 1: Retrieval of parameters using simulated data (A) Example of approximate proliferation posterior of the marked point in (B). (B) Estimated proliferation parameters compared to real parameters from simulated data using 270 initial cells. (C) Cell radius, estimated from the simulations, compared with the real parameter value. (D) Correlation between estimated diffusion and cell radius. (E) The error between estimated, real, and net proliferation from simulations with non-zero death rate. Black bars are 95% CI. (F) Average pair correlation function for simulations with the same net-proliferation but differing death rates. (G) Correlation between estimated diffusion and the real death rate. (A-D) n=1095 simulations. (F) n=500 simulations for each set. (G) n=109 simulations with a death rate of 50% to 90% of the proliferation rate.

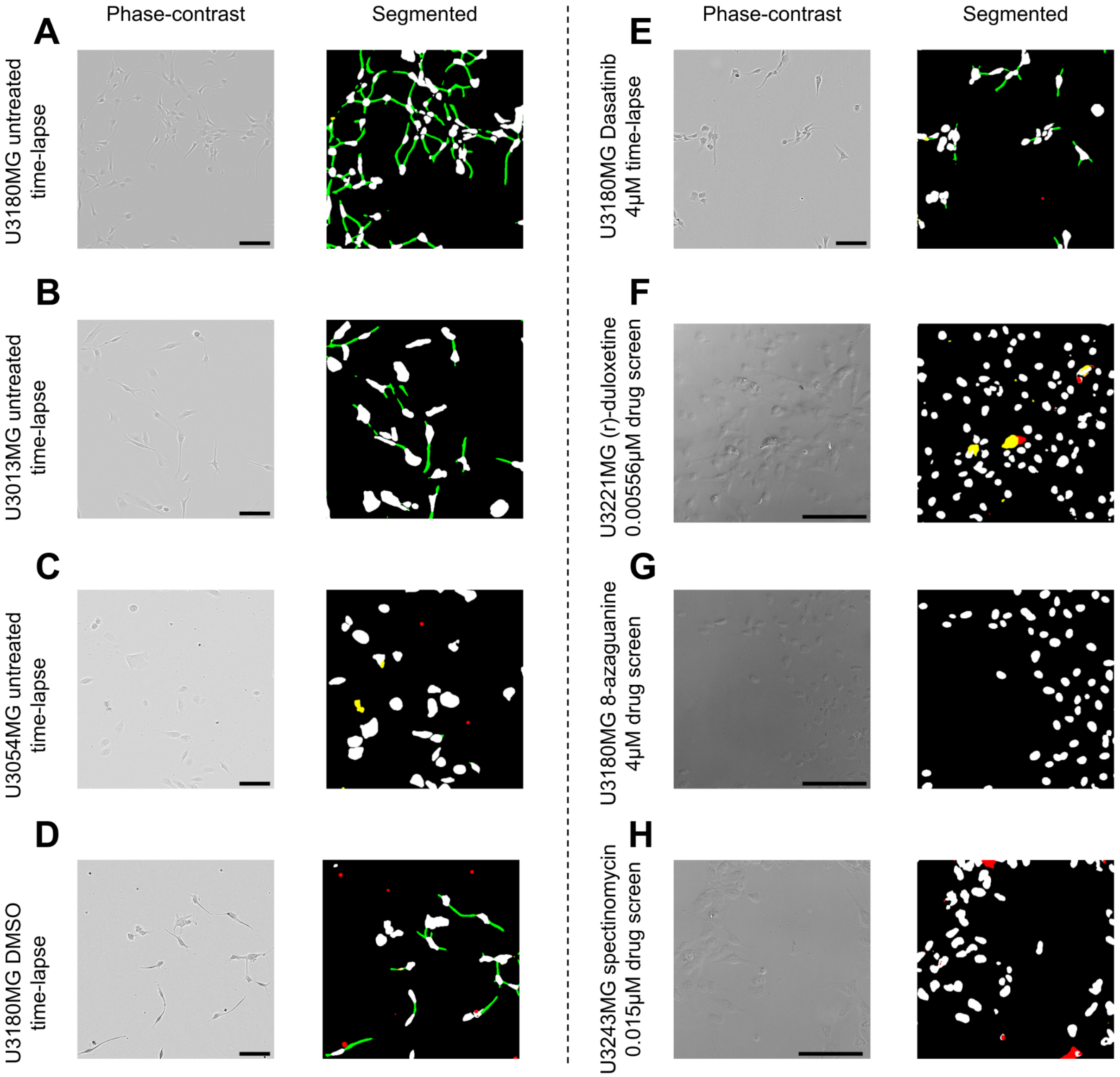

Supplemental Figure 2: Semantic segmentation. Phase-contrast images with the corresponding segmented images from a deep convolutional neural network. (A-D) Images from the time-lapse experiment with no treatments. (E) Time-lapse experiment with treated cells. (F-H) Images from the drug screen. Only the phase-contrast channel is shown. (A-H) Black corresponds to background, white to cells and green to cell protrusions (absent for the drug screen images). Red and yellow to artifacts such as bubbles, cell parts or out of focus areas. The white cell segmentation was used to determine cell positions and size. Touching cells are further separated using water shedding. Images are cropped. Contrast and brightness have been increased. Scale bars are 100 µm.

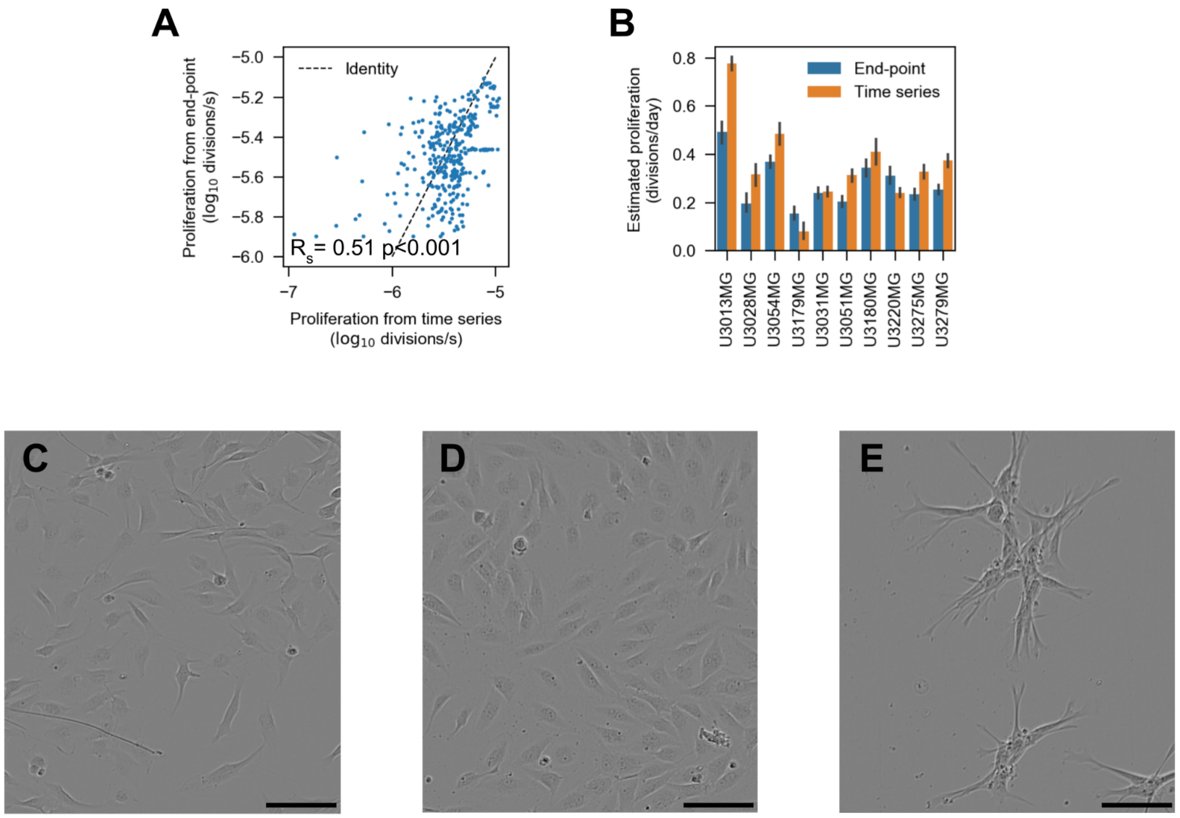

Supplementary Figure 3: Time-lapse imaging of untreated cells (A-B) Estimated proliferation from end-point images compared to estimates from time-series using all time points. Black bars are 95% CI. Stratified by cell culture in (B). n=273 wells. (C-E) *In vitro* phase-contrast images for three different cell cultures (C) U3013MG, (D) U3054MG and (E) U3079MG after 4 days. End-point images looked similar to (C) or (D) for nine of the ten cell cultures. Scale bars are 100µm.

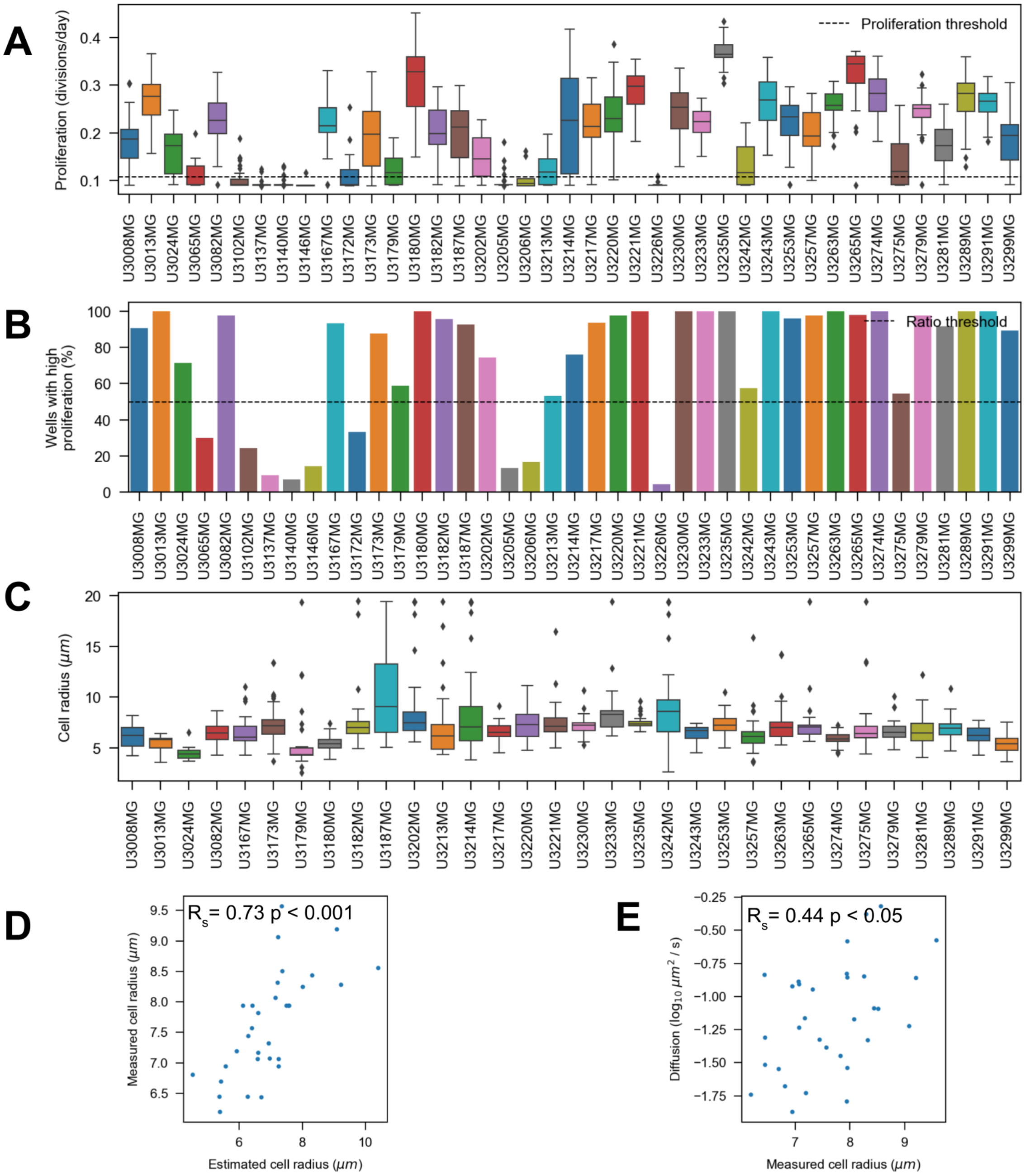

Supplemental Figure 4: Estimated proliferation rates and cell radius for the drug screen experiment. (A) Estimated proliferation rates for different patient-derived glioblastoma cell cultures. The dotted line represents the threshold for trustworthy diffusion estimates. (B) Proportion of wells which are highly proliferative for each PDC. Diffusion is only estimated from wells above the threshold (dotted line). (C) Estimated cell radius for each PDC. (D) Correlation between cell radius estimated from the simulations and measured from the images for the PDCs. (E) Correlation between diffusion estimates and the cell radius as measured from the images for the PDCs. (A-E) n=32 PDCs.

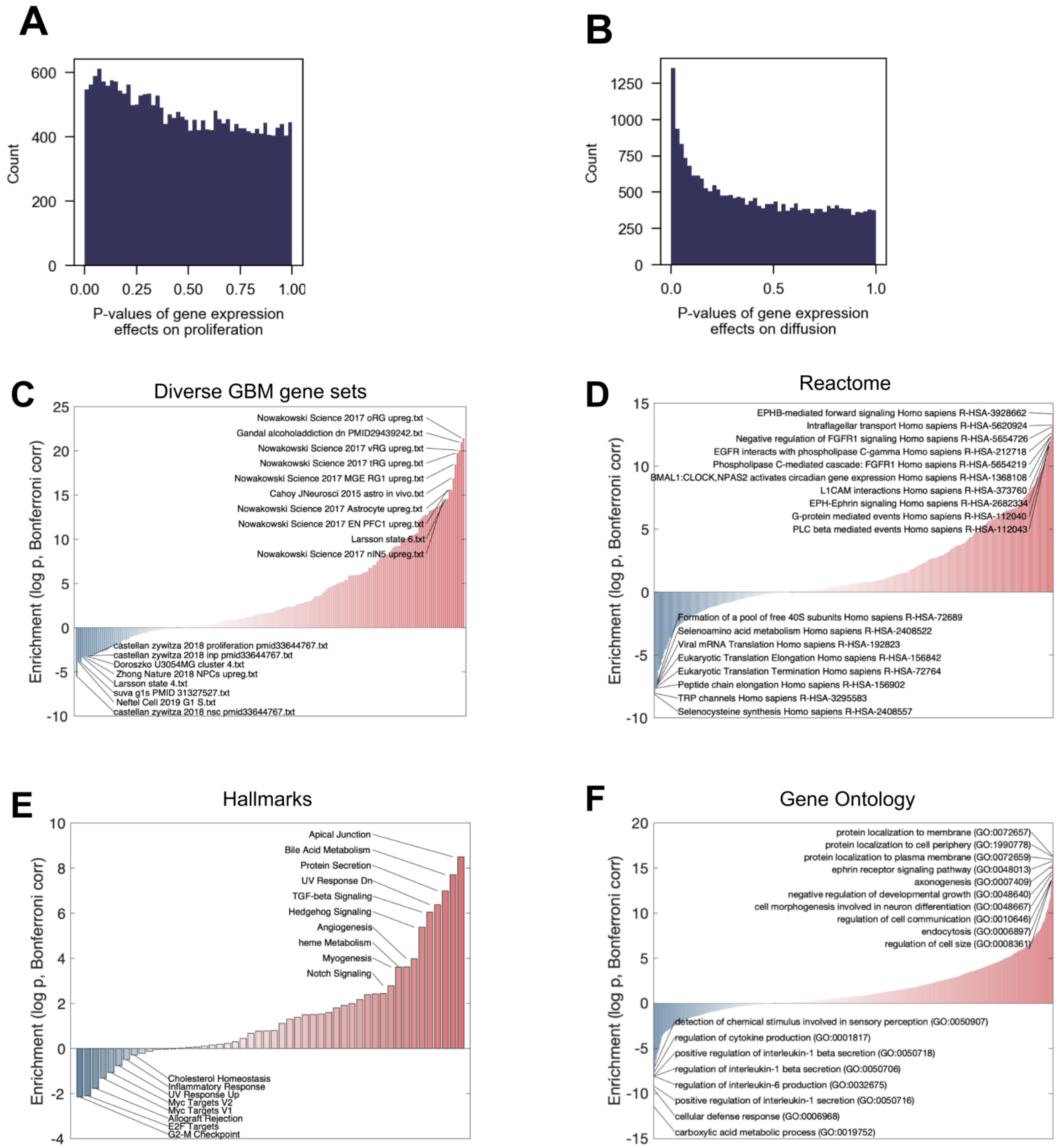

Supplemental Figure 5: Correlation between proliferation, diffusion, gene expression and gene sets. (A-B) P-value histogram between gene expression data and (A), the proliferation rate and (B), the diffusion constant (n=31). (C-F) Enrichment scores between the migration signature and (C) diverse GBM gene sets, (D) Reactome database, (E) MSig database hallmarks and (F) Gene ontology database. The Y-axis represents signed log10 Bonferroni corrected p values. Positive values were positively correlated with the migratory signature.

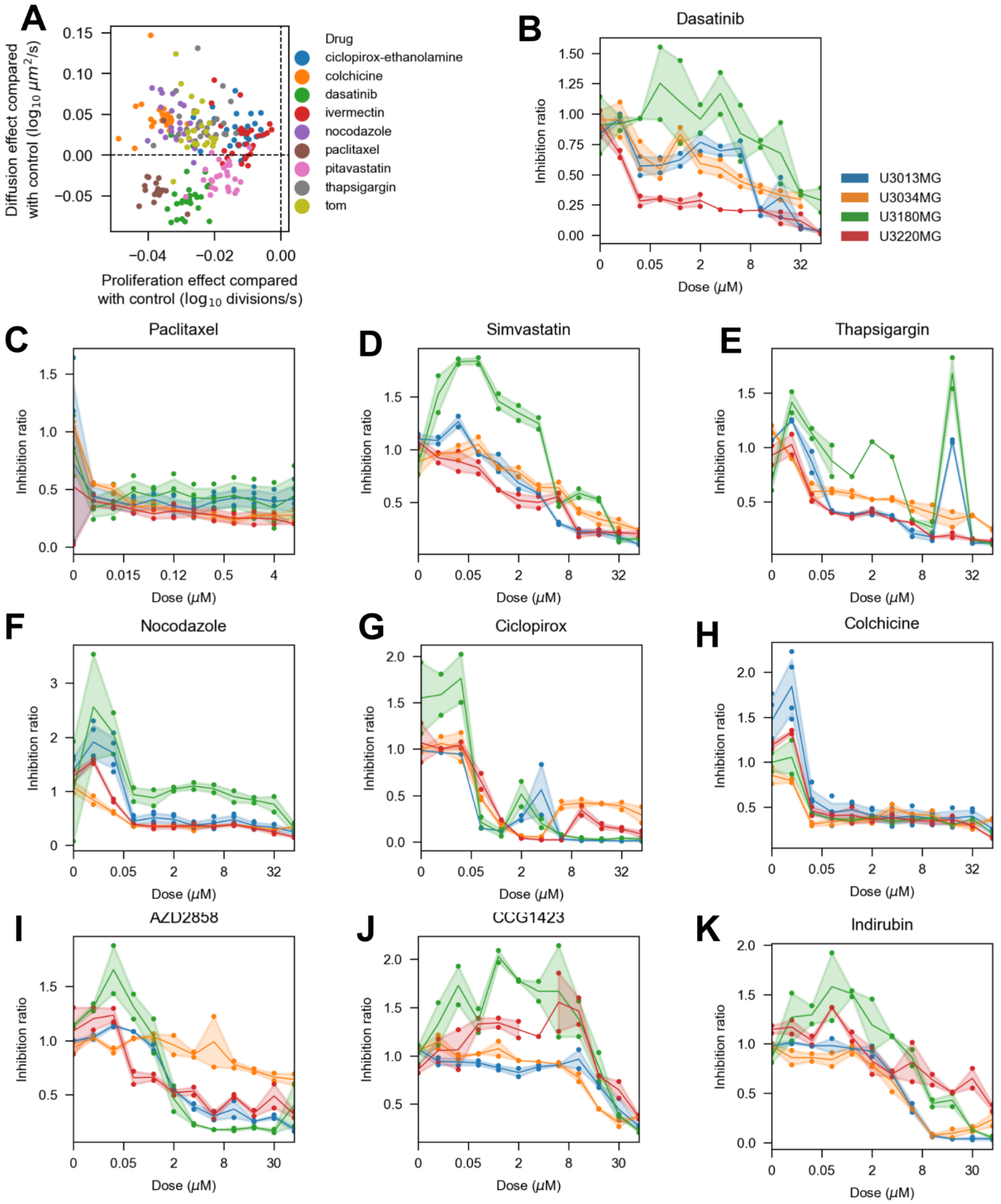

Supplemental Figure 6: Dose response curves for the treated time-lapse data. (A) Cell culture specific diffusion treatment effects for 9 drugs. Points with the same color represent n=32 cell cultures. Colors correspond to different drugs. (B-K) Inhibition ratio of cell count at 72 hours between DMSO control and treated wells for 10 different compounds. Points represent individual wells while the line corresponds to the mean effect. Colors indicate cell culture, and match the legend of Figure 5. Shaded regions indicate 95% confidence intervals.

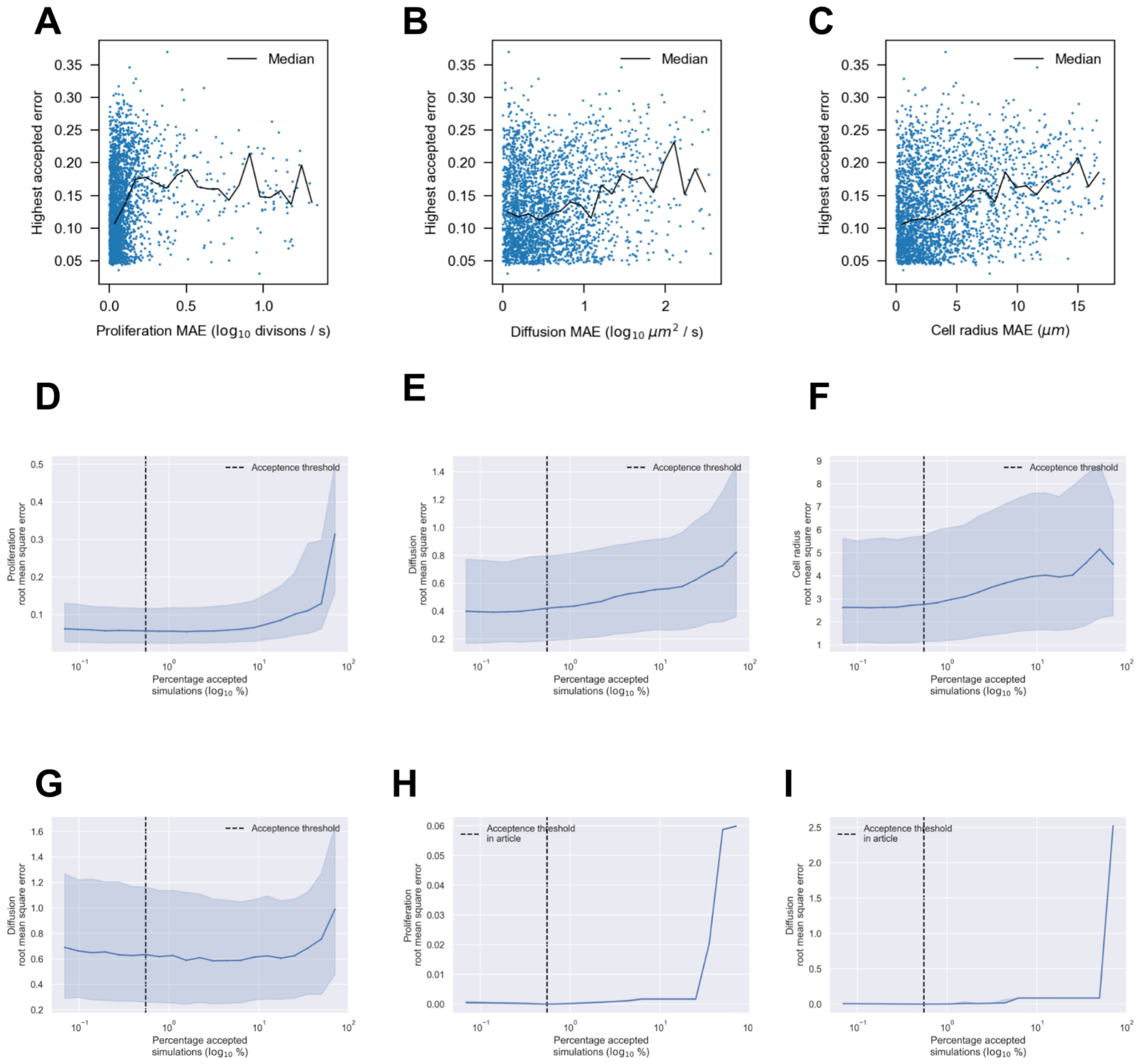

Supplementary Figure 7: Convergence and stability. (A-G) Results based on simulated data. (A-C) Highest accepted ABC-error (distance between simulated and measured cell count and pair correlation function) for each sample, varying with the errors of the parameter estimates. (D-E) Parameter error varying by acceptance ratio. A lower acceptance ratio accepts fewer simulations to build the posterior. (G) Same as (F) but using simulations with a non-zero death rate. (H-I) Results from the drug screen data. Real parameter values unknown. The y-axis shows the difference between the parameter estimates at the reference point (dotted line, acceptance ratio 0.5\%) and other values of the acceptance ratio. Large values indicate that the posterior and parameter estimations are unstable in that region. (D-I) The dotted lines correspond to the acceptance ratio used in the article. The solid lines correspond to the median and the shaded area as the 25% and 75% percentiles. The 25% quantiles in (H-I) are very small and not visible in the figure.
