# Supplementary material for: Inference of glioblastoma migration and proliferation rates using single time-point images
Source: Commun Biol. 2023 Apr 13;6:402. doi: 10.1038/s42003-023-04750-0 (PMC10102065; doi:10.1038/s42003-023-04750-0)
Supplement: Supplementary file 3 — Description of Additional Supplementary Files [file 42003_2023_4750_MOESM3_ESM.pdf]

Description of Additional Supplementary Files

File Name: Supplementary Data

Description: source data behind the graphs in the paper.
