# Supplementary material for: Inference of glioblastoma migration and proliferation rates using single time-point images
Source: Commun Biol. 2023 Apr 13;6:402. doi: 10.1038/s42003-023-04750-0 (PMC10102065; doi:10.1038/s42003-023-04750-0)
Supplement: Supplementary file 5 — Reporting Summary [file 42003_2023_4750_MOESM5_ESM.pdf]

Corresponding author(s): *Double-blind peer review submissions: write DBPR and your manuscript number here instead of author names.*

Last updated by author(s): *YYYY-MM-DD*

Reporting Summary

Nature Portfolio wishes to improve the reproducibility of the work that we publish. This form provides structure for consistency and transparency in reporting. For further information on Nature Portfolio policies, see our Editorial Policies and the Editorial Policy Checklist.

Statistics

For all statistical analyses, confirm that the following items are present in the figure legend, table legend, main text, or Methods section.

n/a	Confirmed	
☐	☒	The exact sample size (*n*) for each experimental group/condition, given as a discrete number and unit of measurement
☐	☒	A statement on whether measurements were taken from distinct samples or whether the same sample was measured repeatedly
☐	☒	The statistical test(s) used AND whether they are one- or two-sided<br>*Only common tests should be described solely by name; describe more complex techniques in the Methods section.*
☐	☒	A description of all covariates tested
☐	☒	A description of any assumptions or corrections, such as tests of normality and adjustment for multiple comparisons
☐	☒	A full description of the statistical parameters including central tendency (e.g. means) or other basic estimates (e.g. regression coefficient) AND variation (e.g. standard deviation) or associated estimates of uncertainty (e.g. confidence intervals)
☐	☒	For null hypothesis testing, the test statistic (e.g. F, t, r) with confidence intervals, effect sizes, degrees of freedom and P value noted<br>*Give P values as exact values whenever suitable.*
☐	☒	For Bayesian analysis, information on the choice of priors and Markov chain Monte Carlo settings
☐	☒	For hierarchical and complex designs, identification of the appropriate level for tests and full reporting of outcomes
☐	☒	Estimates of effect sizes (e.g. Cohen's *d*, Pearson's *r*), indicating how they were calculated

Our web collection on statistics for biologists contains articles on many of the points above.

Software and code

Policy information about availability of computer code

Data collection	no specific software was was used for data generation.
Data analysis	The manuscript uses our own code (github.com/emilrosen/endpoint_cell_diffusion) which runs on python 3.6.2, using the statsmodels 0.12 library

For manuscripts utilizing custom algorithms or software that are central to the research but not yet described in published literature, software must be made available to editors and reviewers. We strongly encourage code deposition in a community repository (e.g. GitHub). See the Nature Portfolio guidelines for submitting code & software for further information.

Data

Policy information about availability of data

All manuscripts must include a data availability statement. This statement should provide the following information, where applicable:

- Accession codes, unique identifiers, or web links for publicly available datasets
- A description of any restrictions on data availability
- For clinical datasets or third party data, please ensure that the statement adheres to our policy

The software is freely distributed via GitHub (above). Image data are available from the authors as states in the manuscript.

Human research participants

Policy information about studies involving human research participants and Sex and Gender in Research.

Reporting on sex and gender	N/A.
Population characteristics	N/A. The patient-derived cell cultures used is reported in a previous publication, Johansson, Krona, Kundu, et al, Cell Reports 2020.
Recruitment	N/A.
Ethics oversight	Patient derived cell collection (described in Johansson, Krona, Kundu, et al, Cell Reports 2020), was approved by the Uppsala regional ethical review board, number 2007/353; informed consent was obtained from all subjects included.

Note that full information on the approval of the study protocol must also be provided in the manuscript.

Field-specific reporting

Please select the one below that is the best fit for your research. If you are not sure, read the appropriate sections before making your selection.

☒ Life sciences ☐ Behavioural & social sciences ☐ Ecological, evolutionary & environmental sciences

For a reference copy of the document with all sections, see nature.com/documents/nr-reporting-summary-flat.pdf

Life sciences study design

All studies must disclose on these points even when the disclosure is negative.

Sample size	The data comprised images from 41 patient-derived cell lines, in which 94 drugs were tested in 11 doses each. (described in Johansson, Krona, Kundu, et al, Cell Reports 2020). Drug testing experiments were carried out over an 11-dose range in at least 2 independent replicates.
Data exclusions	We removed all patient-derived cells where at least 50% of the untreated wells had insufficient proliferation rate. The reason is that in these cases, the method is not applicable since its assumptions aren't met.
Replication	All experiments were done in two independent replicates.
Randomization	This does not apply.
Blinding	This does not apply, as there was no human observer involved.

Reporting for specific materials, systems and methods

We require information from authors about some types of materials, experimental systems and methods used in many studies. Here, indicate whether each material, system or method listed is relevant to your study. If you are not sure if a list item applies to your research, read the appropriate section before selecting a response.

Materials & experimental systems

n/a	Involved in the study	
☒	☐	Antibodies
☐	☒	Eukaryotic cell lines
☒	☐	Palaeontology and archaeology
☒	☐	Animals and other organisms
☒	☐	Clinical data
☒	☐	Dual use research of concern

Methods

n/a	Involved in the study	
☒	☐	ChIP-seq
☒	☐	Flow cytometry
☒	☐	MRI-based neuroimaging

Eukaryotic cell lines

Policy information about cell lines and Sex and Gender in Research

Cell line source(s)	Primary patient-derived cells, see Johansson, Krona, Kundu, et al, Cell Reports 2020.

Authentication	The cell cultures were authenticated by STR profiling, see Primary patient-derived cells, see Johansson, Krona, Kundu, et al, Cell Reports 2020.
Mycoplasma contamination	The cells were checked to exclude mycoplasma, see manuscript.
Commonly misidentified lines (See ICLAC register)	N/A
